# Neonatal Cullen’s Sign: A Distinguishing Feature of Intrauterine Volvulus with Hemorrhagic Ascites

**Published:** 2013-12-01

**Authors:** Federica Pederiva, Angela De Cunto, Giulia Paviotti, Daniela Codrich, Sergio Demarini

**Affiliations:** Institute for Maternal and Child health- IRCCS Burlo Garofolo, Trieste, Italy

A 40-year-old woman presented with reduced fetal movements at 33 weeks’ gestation. Previous antenatal scans had revealed dilated fetal intestine with a suspected diagnosis of intestinal atresia. Cardiotocography revealed fetal bradycardia, fetal ultrasound showed moderate ascites and doppler measurement showed an increase in the middle cerebral artery peak systolic velocity, suggesting fetal acute anaemia. An emergency cesarean section was performed and a live 2070 g male was delivered. Apgar scores were of 9 and 10 at 1 and 5 minutes, respectively. On physical examination at birth the abdomen was distended and tense with a bluish peri-umbilical discoloration of the skin (Fig. 1). Nasogastric aspiration recovered 10 ml of bloody material. The baby was anemic (hemoglobin 11.4 g/dl) and a blood transfusion was given. Abdominal film showed a gastric gas shadow with absent distal gas. Ultrasound examination at 3 hours of life revealed a whirlpool sign and large amount of free fluid mixed with echogenic particles in the abdomen. The newborn underwent laparotomy through transverse supra-umbilical incision and was found to have a 15 cm perforated ileal volvulus distal to a type I atresia of the proximal ileum. After untwisting the volvulus, at the base of it, a secondary ileal atresia was observed. Checking the patency of the distal ileum, other three type I ileal atresias were found. Resection and end-to-end anastomosis of each of the three distal atresias were performed. The necrotic ileal loop involved in the volvulus was resected and the proximal and distal ileum were brought out as a double-barrel ileostomy. Parenteral nutrition and oral feeding were combined with re-feeding of the proximal ostomy effluent into the distal stoma. Five weeks after the initial procedure, the enterostomy was closed.

**Figure:**
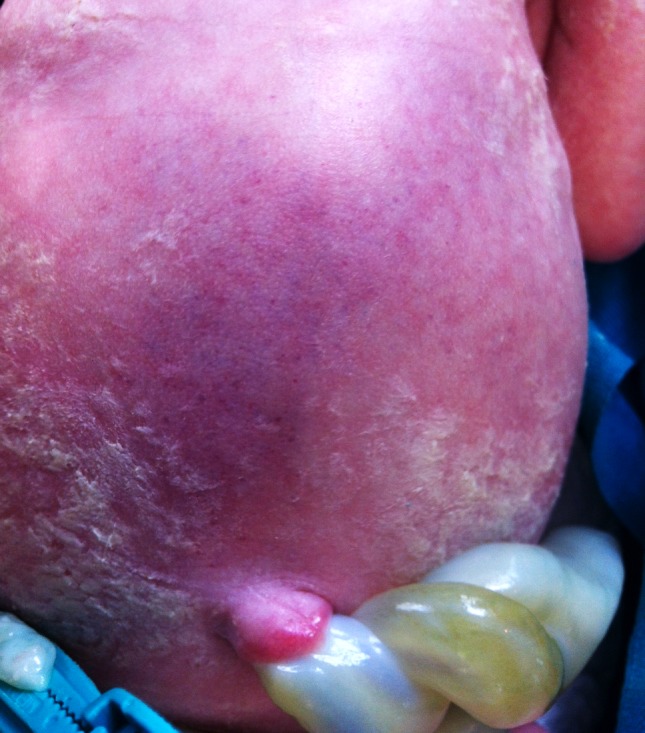
Figure 1: Cullen’s sign

## DISCUSSION

Antenatal diagnosis of congenital midgut volvulus is difficult. It appears on prenatal ultrasound imaging as a twisting of the bowel loops around the mesenteric artery.[1, 2] However, prenatal sonographic diagnosis may be difficult and often missed, so appropriate surgical management might be delayed resulting in ischemic necrosis of the bowel and eventual perforation of the necrotic bowel. Most cases of intra-uterine volvulus are associated with malrotation or congenital anomalies, including intestinal atresia.[3] Detection of the classical whirlpool sign on antenatal scan is very difficult, so the diagnosis of volvulus may be missed. Fetal anemia due to hemorrhagic ascites may develop and acute increased peak systolic velocity in the middle cerebral artery can represent a useful index to assist in deciding for an emergency delivery. Increased peak systolic velocity in the middle cerebral artery and Cullen’s sign were present in our case along with ascites.

Cullen’s sign (a tense abdomen with dark discoloration of the skin due to tracking of hemorrhagic fluid from the retro-peritoneum along the gastrohepatic and falciform ligament to the umbilicus) was originally described after a case of a ruptured extra uterine pregnancy and can be found in many acute adult diseases, including pancreatitis, pancreatic/abdominal trauma, perforated duodenal ulcera and ruptured abdominal aortic aneurysm. In neoonates, it is highly suggestive of volvulus complicated by perforation and clinicians must be aware of the need of emergency surgery when it is present, as delays in diagnosis and intervention contribute to high mortality and morbidity.

## Footnotes

**Source of Support:** Nil

**Conflict of Interest:** None declared

